# Editor's Note on ‘Influence of correct secondary and tertiary RNA folding on the binding of cellular factors to the HCV IRES’

**DOI:** 10.1093/nar/gkad1100

**Published:** 2023-11-11

*Nucleic Acids Research*, Volume 28, Issue 4, 15 February 2000, Pages 875–885, https://doi.org/10.1093/nar/28.4.875

The Editors were alerted in August 2023 about potential issues with Figure 1B: the backgrounds of lanes 2 (5′ dom III (172–227)) and 3 (5′ dom III (145–248)) show a high level of similarity with the backgrounds of lanes 4 (5′ wt) and 5 (5′ S/L).

An Expression of Concern was published in September 2023.

The Editors analysed the figure and noted areas of similarity. One image resulting from that analysis, with the Exclusion filter and Brightness/Contrast adjustments applied, is provided below. Circles of the same colour point to areas of similarity. Orange arrows point to probable splice lines. Green arrows point to areas where a blur tool appears to have been applied.


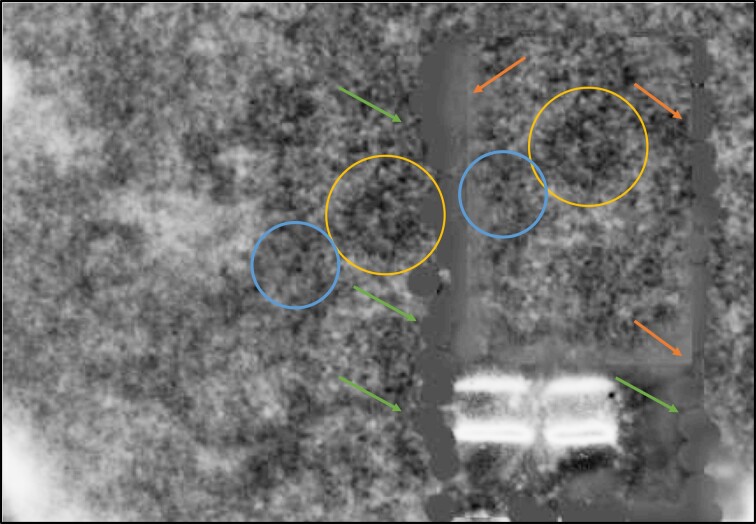


The Editors subsequently contacted the authors, who could not provide the original data because the experiments were conducted over 20 years ago.

The Editors then referred the matter to the authors’ institution for investigation. Ultimately, the institution and authors acknowledge that some splicing did occur in Figure 1B. However, the institution and the authors noted that:

- The ‘masked’ area in the figure is the positive control (lanes 4 and 5).
- There is no evidence of splicing in Figure 1C (*in vitro* transcription and translation of the same mutants). This figure shows that there are no bands above the positive controls, which would correspond to translation products in the mutants above the 25 and 23 kDa compared to controls (lanes 1–3).

The Editors also note that Figures 5B and 5C show essentially the same results as Figures 1B and 1C.

In conclusion, while these issues may not affect the results or conclusion of the study, in the absence of original data, the Editors advise readers to examine Figure 1B with care.

**Julian E. Sale, Barry L. Stoddard**

Senior Executive Editors

